# Supramolecular Hydrogel-Wrapped Gingival Mesenchymal Stem Cells in Cutaneous Radiation Injury

**DOI:** 10.3390/cells11193089

**Published:** 2022-09-30

**Authors:** Shasha Nie, Chunhua Ren, Xin Liang, Hui Cai, Hao Sun, Fengting Liu, Kaihua Ji, Yan Wang, Qiang Liu

**Affiliations:** 1 Institute of Radiation Medicine, Chinese Academy of Medical Sciences & Peking Union Medical College, Tianjin 300192, China; 2 Department of Radiology, Qilu Hospital of Shandong University, Jinan 250012, China; 3 School of Public Health, Tianjin Medical University, Tianjin 300070, China

**Keywords:** cutaneous radiation injury, GMSCs, pY-Gel, EGFR, STAT3

## Abstract

Radiation-induced skin wound/dermatitis is one of the common side effects of radiotherapy or interventional radiobiology. Gingiva-derived mesenchymal stem cells (GMSCs) were indicated to have therapeutic potentials in skin diseases. However, stem cells are prone to spread and difficult to stay in the skin for a long time, limiting their curative effects and application. This study investigated the therapeutic efficacy of Nap-GDFDFpDY (pY-Gel) self-assembled peptide hydrogel-encapsulated GMSCs to treat ^137^Cs γ-radiation-induced skin wounds in mice. The effects were evaluated by skin damage score, hind limb extension measurement and histological and immunohistochemical analysis. In vivo studies showed that pY-Gel self-assembled peptide hydrogel-encapsulated GMSCs could effectively improve wound healing in irradiated skin tissues. In addition, it was found that GMSCs conditioned medium (CM) could promote the proliferation, migration and DNA damage repair ability of skin cells after irradiation in human keratinocyte cell line HaCaT and normal human dermal fibroblasts (HFF). Mechanistically, GMSCs-CM can promote the expression of epidermal growth factor receptor (EGFR), signal transducers and activators of transcription 3 (STAT3) and matrix metalloproteinases (MMPs), suggesting that activation of the EGFR/STAT3 signaling pathway may be involved in the repair of skin cells after exposure to radiations. In conclusion, pY-Gel self-assembled peptide hydrogel-encapsulated GMSCs have a beneficial therapeutic effect on radiation-induced cutaneous injury and may serve as a basis of novel cells therapeutic approach.

## 1. Introduction

Ionizing radiation (IR) is widely used in medical and industrial fields. Although exposure to extreme radiation is rare, accidents like the Chernobyl and Fukushima nuclear power plants are still possible [1]. Accidental or therapeutic exposure to high-dose IR can cause severe damage to living tissues. Tissues that proliferate at a higher rate and are adequately oxygenated are more susceptible to the effects of radiation including on organ systems, such as the hematopoietic, gastrointestinal and skin systems [2,3]. The skin is a continuously renewing organ system containing rapidly proliferating and maturing cells [4,5]. Therefore, the skin is highly susceptible to radiation damage [2,6]. Cutaneous radiation injury (CRI) occurs in about 95% of patients receiving radiation therapy for cancer [7,8]. CRI can significantly reduce the quality of life in patients after radiotherapy. Despite substantial improvements in radiological technology, radiation-induced skin damage is still a cause of concern. The lack of drugs to treat severe radiation-induced skin injuries has prompted further research into the development of better treatment options. In recent years, cell-based therapies have become a promising approach for the treatment of chronic difficult-to-heal wounds. Mesenchymal stem cells (MSCs) have been proven to be the frontier of therapeutic strategy development for various diseases [3].

MSCs are multipotent stem cells found in multiple tissues, such as bone marrow, adipose tissue, dermis, brain and spleen [9,10]. Studies have determined that MSCs achieve their regenerative potential in skin trauma by promoting immunosuppression [11,12], angiogenesis [13,14], anti-apoptosis [15], differentiation [16,17] and proliferation [18], as well as alleviating fibrosis and hypertrophic scars [19,20,21]. MSCs per se are not the only elements used in these studies; MSC-derived products have shown paracrine beneficial effects to present a therapeutic effect for wound healing and the regeneration of tissue [22]. It has been reported that MSC-derived extracellular vesicles have immunosuppressive and immunomodulatory properties, as well as the ability to activate the angiogenesis, proliferation, migration and differentiation of endothelial cells, fibroblasts and keratinocytes, which are accelerated by paracrine activity in chronic skin ulcers, healing and decreasing scar formation, and have positive effects [23]. MSCs have been found in various injury models with a strong regenerative ability and seem to be a potential treatment for radiation-induced skin injury [3]. The gingiva is a unique soft tissue that serves as a biological mucosal barrier covering the oral side of the maxilla and mandible. Unlike scar formation in the skin, wound healing in the gingival and oral mucosa is characterized by reducing inflammation significantly, rapid epithelial regeneration and scarless fetal healing [24,25]. Gingiva-derived MSCs (GMSCs) have a unique origin from the neural crest and show the ability of self-renewal, multipotent differentiation and immune regulation in vitro and in vivo [26,27]. A growing body of evidence shows that GMSCs have stable phenotypes and can maintain a normal karyotype and telomerase activity in long-term culture. In addition, GMSCs are derived from healthy or hyperplastic and (or) inflamed gingiva tissues in humans, and they are not carcinogenic [28,29]. Recent studies showed that GMSC-derived exosomes contain many cytokines to facilitate wound healing in the gingiva [30]. As gingival tissue is abundant and readily available, GMSCs may be superior to bone marrow mesenchymal stem cells in cell therapy for regenerative medicine [28]. Thus, GMSCs could also play a key role in the treatment of radiation-induced skin injuries.

Hydrogel biomaterials have been widely used in tissue engineering to provide an extracellular microenvironment for tendon regeneration by GMSCs [31]. Moreover, it has been reported that the encapsulated MSCs remain viable within the hydrogel, accelerating wound healing via enhancing angiogenesis and suppressing local proinflammatory cytokines [32]. Nap-GDFDFDY (Y-Gel) is a molecular hydrogel of co-assembled peptide gel and protein, which can optimize the humoral immune response of mice through various routes of administration. It has good biocompatibilities against mice immune cells, such as mononuclear macrophages and spleen cells, a prerequisite for biomaterials in biomedical applications [33,34]. Phosphorylated Y-Gel can form the supramolecular hydrogel Nap-GDFDFpDY (pY-Gel) in the presence of Ca^2+^, which is biodegradable and biocompatible in vivo, could not only prevent the rapid diffusion of antigens from injection sites, but also adhere well to tissue [35,36], which seems to be an advantageous characteristic for wound healing. It might be another promising scaffolding material for the encapsulation of stem cells that effectively delivers patient-derived dental-derived MSCs.

In this study, the skin of C57BL/6J mice was exposed to ^137^Cs γ-ray source to establish a mouse model of radiation-induced skin damage. After subcutaneous injections of pY-Gel self-assembling peptide hydrogels for GMSCs, we evaluated the therapeutic effects on radiation-induced skin injury in mice and further investigated the possible mechanism by in vitro cell experiments.

## 2. Materials and Methods

### 2.1. Cell Culture and Preparation of Conditioned Medium

HaCaT and HFF cells were kindly provided by Stem Cell Bank, the Chinese Academy of Sciences. HaCaT was cultured in RPMI 1640 (HyClone, Logan, UT, USA) supplemented with 10% fetal bovine serum (FBS; Gibco, Carlsbad, CA, USA). HFF was cultured in DMEM (HyClone, Logan, UT, USA) supplemented with 10% fetal bovine serum (FBS; Gibco, Carlsbad, CA, USA). GMSCs were kindly donated by Beijing Taisheng Biotechnology Co., Ltd. (Beijing, China). GMSCs were cultured in α-MEM (HyClone, Logan, UT, USA) supplemented with 10% fetal bovine serum (FBS; Gibco, Carlsbad, CA, USA). To avoid changes in cell behavior due to prolonged culture time, only GMSCs at passages P3–P6 were used in this study.

GMSCs were cultured to 80% confluency in 10% FBS complete medium. Then, the medium was replaced by fresh culture medium, and the cells were cultured for another 48 h at 37 °C under 5% CO_2_. The supernatants of the GMSCs were collected and then passed through 0.45 μm micrometer filters to obtain conditioned medium. All conditioned mediums were subpackaged and stored at −80 °C.

### 2.2. Animals and Radiation-Induced Skin Injury Model

Male C57BL/6J mice were purchased from Beijing Vital River Laboratory Animal Technology Co., Ltd. (Beijing, China). All mice had ad libitum access to food and water and were kept in specific-pathogen-free (SPF) animal rooms at the laboratory animal center of the Institute of Radiation Medicine, Chinese Academy of Medical Science. Age-matched 6- to 8-week-old male mice from the same background were used in all experiments. All animal experiments were reviewed and approved by the Animal Ethical and Welfare Committee (AEWC).

The mice were randomly divided into five groups the day before exposure to IR. The mice were anesthetized and the left hind limb skin was exposed to 50 Gy of ^137^Cs γ-ray at a dose rate of 0.890 Gy/min using a Nordion Gammacell 40 whole animal irradiator (BEST Theratronics Ltd., Ottawa, ON, Canada).

### 2.3. Nap-GDFDFpDY (pY-Gel) Self-Assembling Peptide Hydrogels

The pY-Gel was provided by Dr. Liu Jianfeng (Chinese Academy of Medical Sciences and Institute of Radiation Medicine of Peking Union Medical College, Tianjin, China). pY-Gel powder was dissolved in PBS at 3 mg/mL, mixed well, pH was adjusted to 7.3–7.4, alkaline phosphatase 1 U/100 μL was added and the pY-Gel supramolecular hydrogel was placed on ice for later use.

### 2.4. Transplantation of GMSCs in Living Mice and Wound Assessment

In all groups, an identical dose of GMSCs (5 × 10^5^ GMSCs/mouse) was used throughout this study. The mice used in the in vivo GMSCs transplantation experiment were divided into five groups (*n* = 5 for each group), namely NO-IR, IR+ PBS, IR+ pY-Gel, IR+ GMSCs and IR+ pY-Gel+ GMSCs. The GMSCs were encapsulated into the pY-Gel and then injected into the injury sites with four injections per mouse at 24 h after exposed to IR.

Radiation-induced skin reactions were assessed by double-blind scoring after exposure to IR. The degree of cutaneous toxicity was evaluated every two days, based on the skin score criteria of a 5-point scoring system [37]. The irradiated area of each mouse was photographed with a digital camera every five days. Limb extension was measured at baseline 30 days after IR. Briefly, the maximum extension of the irradiated (left) and non-irradiated (right) limbs of the mice were measured [38]. Then, the ratio of the extension of the irradiated side to the stretching of the non-irradiated side was calculated, with a value of one representing equivalent extension.

### 2.5. Histological Examination

The mice were euthanized 30 days after exposure to IR. The skin was fixed in a formaldehyde solution (# 50-00-0; Damao Chemical Reagent Factory, Tianjin, China) and prepared for histological analysis. Samples were dehydrated, embedded in paraffin, cut into 5 μm micrometer slices using a microtome and mounted on glass slides. Samples were stained with hematoxylin and eosin (H&E; # G1080 and # G1100; Solarbio, Beijing, China) and Masson’s Trichrome (# G1345; Solarbio). Images were captured using a Zeiss Axio Lab.A1 upright microscope (Carl Zeiss MicroImaging GmbH, Jena, Germany).

To detect the tissue proliferation marker Ki67, sections were also immunostained. Briefly, sections were incubated with rabbit anti-Ki67 primary antibody (ab15580; Abcam, Cambridge, UK). Slides were subsequently stained with biotinylated goat anti-rabbit secondary antibody (# PV-9000; ZSGB-BIO, Beijing, China). Reaction sites were visualized with DAB (ab64238; Abcam), and the slides were counterstained with hematoxylin. The number of Ki67-positive cells was assessed by random selection of a field of view per sample using a Zeiss Axio Lab.A1 upright microscope.

### 2.6. Western Blot Analysis

Cells or tissues were lysed in RIPA buffer (# R0010; Solarbio) with PMSF (# P0100; Solarbio) and protease inhibitors (04693132001; Roche AG, Basel, Switzerland), and proteins were quantified using a bicinchoninic acid (BCA) Protein Assay Kit (# P0010; Beyotime Institute of Biotechnology, Shanghai, China). For Western blotting of lysate proteins, 20 μg of proteins were separated by 8 or 10% sodium dodecyl sulfate–polyacrylamide gel electrophoresis (SDS-PAGE) and transferred to polyvinylidene fluoride (PVDF) membranes (IPVH00010; Merck Millipore KGaA, Darmstadt, Germany). The membranes were blocked with 5% Difco Skim Milk (232100; BD Biosciences, San Jose, CA, USA) for 2 h, followed by overnight incubation with the corresponding primary antibodies. Subsequently, the membranes were incubated at room temperature for 1 h with a horseradish peroxidase (HRP)-conjugated secondary antibody. Immunoreactive proteins were detected using SuperSignal West Pico PLUS Chemiluminescent Substrate (34580; Thermo Fisher Scientific Inc., Waltham, MA, USA) and ChemiDoc™ MP Imaging System (Bio-Rad Laboratories, Hercules, CA, USA). The primary antibodies used were as follows: β-tubulin, GAPDH (ProteinTech Group Inc, Rosemont, IL, USA), cyclin A (Santa Cruz Biotechnology, Santa Cruz, CA, USA), STAT3, pSTAT3, c-Myc, Ki67 (Abcam), EGFR, pEGFR and γH2AX (Cell Signaling Technology Inc., Danvers, MA, USA).

### 2.7. Quantitative Real-Time PCR Analysis

Total RNA was extracted using TRIzol reagent (15596018; Invitrogen, Carlsbad, CA, USA), and the concentration and quality of the RNA were determined by spectrophotometry using a NanoDrop 8000 spectrophotometer (Thermo Fisher Scientific Inc.). cDNA synthesis was performed using the PrimeScript™ RT Master Mix (Perfect Real Time; Cat# RR036A, Takara Bio Inc., Otsu, Japan) following the manufacturer’s protocol. The real-time PCR was performed using a BlasTaq™ 2× qPCR MasterMix (G891; Applied Biological Materials (abm) Inc., Richmond, BC, Canada), according to the manufacturer’s protocol. All data were normalized to the control using GAPDH as an internal control. The primers for the qPCR are listed in Table S1.

### 2.8. Proliferation Assay

Cells were seeded in 96-well plates at a density of 5 × 10^3^ cells/well in triplicate and incubated at 37 °C. These cells were treated with fresh medium supplemented with GMSCs-CM in the ratio 1:1 and exposed to irradiation 4 and 8 Gy immediately, followed by incubation for 24, 48 and 72 h, respectively. Cell proliferation was evaluated using the Cell Counting Kit-8 (CCK-8; # MA0218; Meilunbio, Dalian, China) assay. Briefly, 10 μL of CCK-8 reagent was added to each well, and plate was incubated for 2 h. Absorbance readings were measured at 450 nm on a Synergy HT microplate spectrophotometer (BioTek Instruments Inc., Winooski, VT, USA).

### 2.9. Colony Formation Assay

HaCaT cells were seeded in 6-well plates in triplicate at a density of 1000 cells/well, treated with fresh medium supplemented with GMSCs-CM in the ratio 1:1 and exposed to the indicated doses of ^137^Cs γ-radiation (0.890 Gy/min) 1, 2 and 4 Gy immediately. The cells were then incubated for about 10 days, changing the medium every 3 days. Finally, the cells were stained with crystal violet (# C8470; Solarbio), and colonies containing more than 50 cells were counted.

### 2.10. Scratch Wound Healing Assay

Cell migration was assessed by a wound healing assay. HaCaT cells were grown in 6-well plates until they reached a confluence of around 90–100%. A scratch was made in the cell monolayer in middle of the well with a sterilized 10 μm microliter disposable pipette tip, and a photograph was taken. These cells were treated with serum-free medium supplemented with GMSCs-CM in the ratio 1:1 and immediately exposed to IR 8 Gy; images of the scratched area were captured 12 h later. The area between the two edges of the wound was measured using the Image J software (National Institutes of Health (NIH), Bethesda MD, USA). The closed area of the wound was calculated using the following formula: migration area (%) = (S_0_−S_n_)/S_0_ × 100, where S_0_ denotes the initial wound area and Sn denotes the remaining wound area at the measurement point.

### 2.11. Transwell Migration Assay

Cells were suspended in serum-free medium and seeded at 6 × 10^4^ cells/well into the upper chambers of Transwell 24-well plates (3422; Corning Inc., Corning, NY, USA) with 8 μm micrometer pore membranes. Then, complete fresh medium supplemented with or without GMSCs-CM was added to the lower chambers in the ratio is 1:1, irradiating 8 Gy immediately. The cells were incubated at 37 °C for 12 h, and non-migrating cells were removed with cotton swabs. Migrated cells of the lower surface were stained with 0.5% crystal violet for 15 min. Then, stained cells were visualized under a microscope and manually counted.

### 2.12. Immunofluorescence and Quantitative Analysis of Individual Cells

Cells were cultured on glass coverslips and treated with fresh medium supplemented with GMSCs-CM in the ratio 1:1 before exposure to IR 8 Gy. Cells were fixed with 4% paraformaldehyde (# P1110; Solarbio) for 15 min and permeabilized with 0.3% Triton-X 100 (# T8200; Solarbio), and non-specific binding was blocked with 1% Albumin Bovine V (A8020; Solarbio) in PBS for 1 h at room temperature. Fixed cells were incubated with the corresponding primary antibodies at 4 °C overnight, followed by 1 h at room temperature with the appropriate secondary antibody. Then, nuclei were stained with Vectashield Mounting Medium with DAPI (H-1200; Vector Laboratories, Burlingame, CA, USA). Images were captured using an EVOS fluorescent microscope (Advanced Microscopy Group (AMG), Bothell, WA, USA) and processed using Adobe Photoshop CS6 (Adobe Inc., San Jose, CA, USA). The numbers of foci in individual cells were counted, and the data were plotted using the GraphPad Prism 8 software (GraphPad Software Inc., San Diego, CA, USA). The primary antibody used was γ-H2AX (05-636; Merck Millipore KGaA). Secondary antibody used was Cy3-conjugated goat anti-mouse IgG (H+L) (SA00009-1; ProteinTech Group Inc.).

### 2.13. Cell Cycle Analysis

The cell cycle distribution was analyzed after treating the cells with fresh medium supplemented with GMSCs-CM in the ratio 1:1 and followed by exposure to IR 8 Gy immediately. Cells were fixed with cold 70% ethanol for 2 h at 4 °C. Afterwards, cells were stained with propidium iodide (PI) staining buffer (50 µg/mL PI, 4 mM sodium citrate buffer and 10 µg/mL RNase A) and incubated at 37 °C for 15 min in the dark. At least 10,000 cells per condition were counted by flow cytometry. The relative distribution of cells over the different cell cycle phases was analyzed using the FlowJo 7.6 software (Tree Star Inc., Ashland, OR, USA).

### 2.14. RNA Interference

After cells were grown to 50–60% confluence, HaCaT cells were transfected with small interfering RNAs (siRNAs) (GenePharma Co., Ltd., Shanghai, China) using Lipofectamine™ RNAiMAX Transfection Reagent (13778150; Invitrogen), following the manufacturer’s instructions. Forty-eight hours after transfection, cells were treated with fresh medium supplemented with or without GMSCs-CM in the ratio 1:1 and immediately exposed to IR, 4 Gy and 8 Gy. The siRNAs for RNA interference are listed in Table S2.

### 2.15. Statistical Analysis

All data are expressed as the mean ± standard deviation. Statistical comparisons were made by analysis of variance (ANOVA), for multiple comparisons, and Student’s *t*-test for two sample comparisons, using the SPSS 25.0 software (IBM Corporation, Armonk, NY, USA). ns, not significant *p* > 0.05, * *p* < 0.05, ** *p* < 0.01 and *** *p* < 0.001 were used in this study to show statistical significance.

## 3. Results

### 3.1. Stem Cell Encapsulation by Nap-GDFDFpDY (pY-Gel) Self-Assembling Peptide Hydrogels

We expanded the isolated primary GMSCs to the P_3_-P_6_ generation in vitro and found that the cells were all fusiform with abundant cytoplasm and good refractive properties (Supplementary Figure S1A–D). A translucent hydrogel was formed by adding alkaline phosphatase to pY-Gel (Supplementary Figure S1E–G), and GMSCs (5 × 10^5^ cells/100 μL) were encapsulated in it (Supplementary Figure S1H). After successfully encapsulating GMSCs with the pY-Gel, the encapsulated GMSCs were injected into mice (100 μL/mouse, divided into four injection sites).

### 3.2. Evaluation of Healing Quality of the CRI

**Figure 1:**
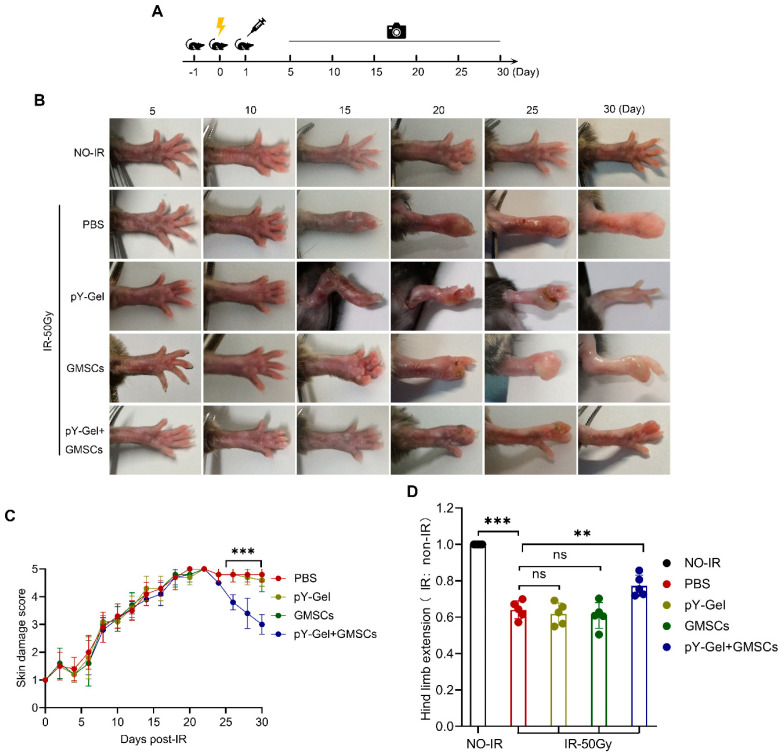
Serial response of mice treated with pY−Gel and GMSCs. (**A**) The flow chart of the development and treatment of a mouse model of CRI. (**B**) Representative images of the left hind limb 5–30 days post-irradiation. (**C**) A 5-point scoring system was used to double-blindly evaluate the degree of skin damage every two days. (**D**) Assessment of hind limb extension. *n* = 5 animals. Data are expressed as the mean ± standard deviation. Statistical comparisons were made by ANOVA for multiple comparisons. ns, not significant *p* > 0.05, ** *p* < 0.01 and *** *p* < 0.001.

In this study, to determine whether GMSCs can be used alone or in combination with other therapies to promote the repair of CRI, a mouse model of CRI (^137^Cs γ-ray, 50 Gy) was established. The treatment was performed within 24 h of the exposure to IR, and the mice were sacrificed 30 days after exposure to IR to obtain tissues for analysis. The flow chart of the experimental procedure is as shown in Figure 1A.

Erythema, alopecia and edema gradually appeared in the left hind limb skin of mice 5 to 10 days after irradiation; exudation, ulceration and necrosis gradually appeared in 10–15 days; the CRI score was the highest at 22 ± 2 days (Figure 1B,C). The results revealed that the mice in the pY-Gel+ GMSCs group exhibited the least skin damage, the latest peak of damage and the shortest skin damage repair time. To assess the degree of skin tissue fibrosis in the mice, we measured the passive leg elongation of mice 30 days after exposure to IR. The results showed that the passive leg elongation of the mice in the IR group was shorter than that of the mice in the control group, but the degree of shortening was considerably alleviated by the treatment with pY-Gel + GMSCs (Figure 1D). The results indicate that the pY-Gel self-assembling peptide hydrogels for GMSCs have a certain therapeutic effect on CRI in mice, delay the appearance of skin damage, reduce the degree of damage and promote skin damage repair.

**Figure 2:**
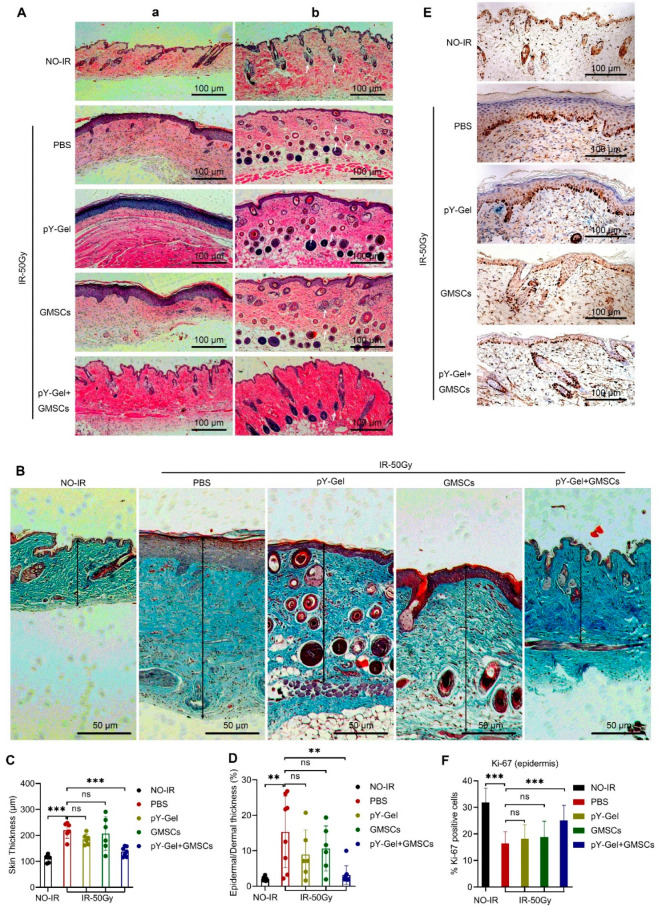
Evaluation of the healing quality of CRI. (**A**) H&E staining images of limb lesions in mice with radiation injury at 30 days post-treatment. The white arrows point to the sebaceous glands in the dermis. (**B**) Masson’s trichrome staining of wound sections. The black arrows indicate the skin thickness. (**C**,**D**) Skin thickness is measured from the dermal–subcutaneous interface to the outer surface of the epidermis and the ratio of epidermis/dermis. (**E**,**F**) Representative immunohistochemical images and quantitation of Ki67 in skin epidermis from non-irradiated mice and irradiated mice 30 days after irradiation. Scale bars, 100 μm. *n* = 5. Data are expressed as the mean ± standard deviation. Statistical comparisons were made by ANOVA for multiple comparisons. ns, not significant *p* > 0.05, ** *p* < 0.01 and *** *p* < 0.001.

In the irradiated group, H&E staining of the skin tissues showed thickened epidermis, irregular cell arrangement, parakeratosis, occasional epidermis absence, lack of skin appendages in the dermis and numerous inflammatory cell infiltrations (Figure 2A in a). In addition, we also found occasional excessive proliferation of sebaceous glands in the dermis, and the cytoplasm was filled with lipid droplets (Figure 2A in b). In contrast, in the pY-Gel+ GMSCs group, the thickening of the epidermal layer was reduced and a relatively regular cell arrangement was observed. Moreover, there were more and more regular skin appendages in the dermis, inflammatory cell infiltrations were relatively rare and the skin histological features tended to be normal (Figure 2A). In addition, we have verified in skin tissue and cells that the expression of IL6, IL1β and TNFα was reduced with treatment, suggesting that inflammatory response is decreased (Supplementary Figure S2). We also performed Masson trichrome staining and measured the skin thickness to determine the ratio of epidermis/dermis. The results showed that more collagen fibers deposited in the skin tissue of the mice in the irradiated group than in the non-irradiated group. Instead, in the pY-Gel+ GMSCs group, the deposition of collagen fibers was significantly reduced. In addition, the measurements of the skin thickness and the ratio of epidermis/dermis revealed that the skin tissue of the mice were significantly thickened after exposure to IR. In contrast, the skin thickness of mice treated with pY-Gel + GMSCs tended to be normal. The measurement of the ratio of epidermis/dermis suggested that the skin thickening was mainly distributed in the epidermis, and the skin of the mice in the pY-Gel+ GMSCs group tended to show normal epidermal thickness (Figure 2B–D). These results showed that exposure to IR can cause skin tissue damage in mice, and pY-Gel self-assembling peptide hydrogels for GMSCs can effectively reduce the degree of radiation-induced skin fibrosis and improve the healing quality.

We also performed Ki67 staining of epidermal cells by immunohistochemistry. The results showed that the proportion of Ki67-positive cells in the epidermal layer of the skin of the irradiated mice was significantly reduced compared with that in the non-irradiated group. In contrast, the pY-Gel+ GMSCs group could partially mitigate the reduction in Ki67-positive cells (Figure 2E,F). Accordingly, these results showed that exposure to IR can decrease the normal proliferation capacity of epidermal cells in the skin tissues. Treatment of CRI with pY-Gel self-assembling peptide hydrogels for GMSCs can partially restore the proliferation capacity of epidermal cells and improve wound healing.

### 3.3. GMSCs Promote the Proliferation and Migration of Skin Cells

**Figure 3:**
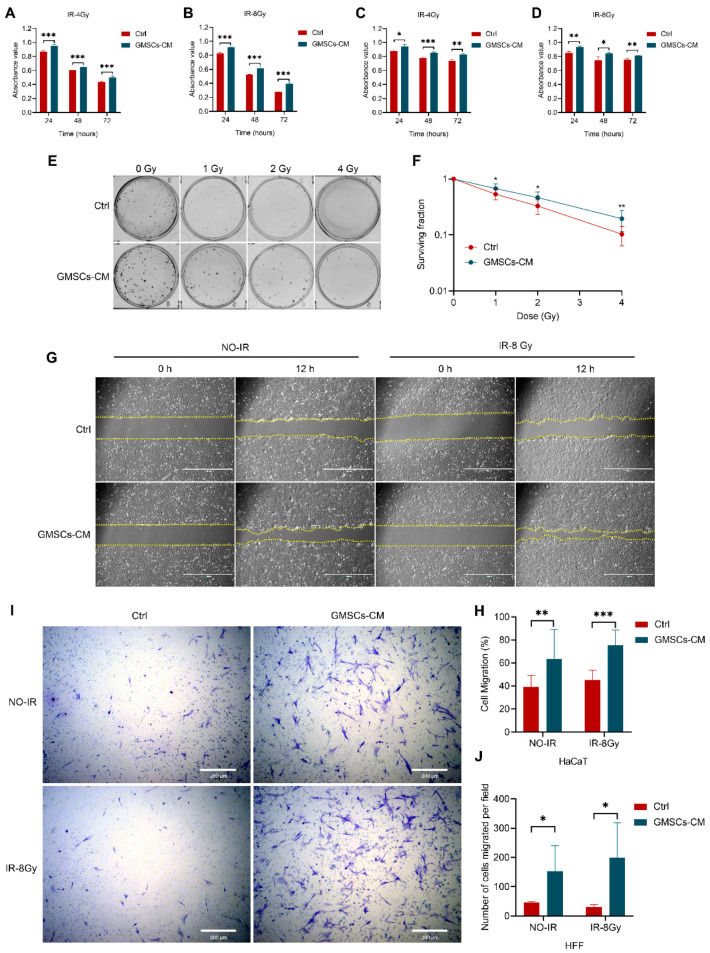
GMSCs promote the proliferation and migration of skin cells. (**A**,**B**) CCK-8 assay of HaCaT cell proliferation. (**C**,**D**) CCK-8 assay of HFF cell proliferation. (**E**,**F**) HaCaT cells were exposed to the indicated IR dose of 0, 1, 2 or 4 Gy and cultured for 10 days. Colonies with more than 50 cells were counted. (**G**) GMSCs-CM promoted HaCaT cells migration according to the analysis by the scratch wound assay. Scale bars, 1000 μm. (**H**) Quantitative analysis of the cell migration rates in (**G**). (**I**,**J**) The migration ability of HFF cells treated with GMSCs-CM was measured by the Transwell assay. Representative results from one of three independent experiments are shown. Data are expressed as the mean ± standard deviation. Statistical comparisons were made by a two-sample Student’s *t*-test, using the SPSS 25.0 software (IBM Corporation, Armonk, NY, USA). * *p* < 0.05, ** *p* < 0.01 and *** *p* < 0.001.

Recent studies have shown that MSCs can secrete various pro-proliferation, anti-inflammatory and pro-angiogenic factors or chemokines to promote the survival and proliferation of skin cells, thereby accelerating wound repair [16,17,18]. To evaluate if the GMSC’s effect seen in vivo was related to factors secreted from the cells, we decided to perform in vitro assays using GMSCs-CM. The irradiation doses used in the assays were established based on previous studies [37,39]. First, we measured the proliferation capacity of skin cells using the CCK-8 assay. The results showed that the proliferation capacity of HaCaT cells was significantly reduced after exposure to IR of 4 and 8 Gy for 24, 48 or 72 h, respectively. Instead, compared with the control group, the cell proliferation capacity of the cells in the GMSCs-CM group was increased (Figure 3A,B). Similar results were obtained with HFF cells (Figure 3C,D). In addition, we also tested the colony formation ability of HaCaT cells. The results revealed that the colony formation rate of HaCaT cells was significantly reduced after exposure to IR in a dose-dependent manner. On the other hand, compared with the control group, GMSCs-CM promoted cell colony formation (Figure 3E,F). The above experimental results indicated that GMSCS-CM promoted the proliferation of skin cells.

The migration of epidermal keratinocytes is an important step in skin wound healing [40]. Thus, we hypothesized that GMSCs might induce cell migration, while promoting the proliferation of skin cells. The results showed that GMSCs-CM treatment significantly increased the migration rate of HaCaT cells. Specifically, GMSCs-CM treatment enhanced cell motility (Figure 3G,H). Transwell migration assay further confirmed that GMSCs-CM treatment promoted the migration ability of HFF cells (Figure 3I,J). The above experimental results demonstrated that GMSCS-CM can promote the migration of skin cells.

### 3.4. GMSCs Enhance the Ability of Skin Cells to Repair DNA Damage

**Figure 4:**
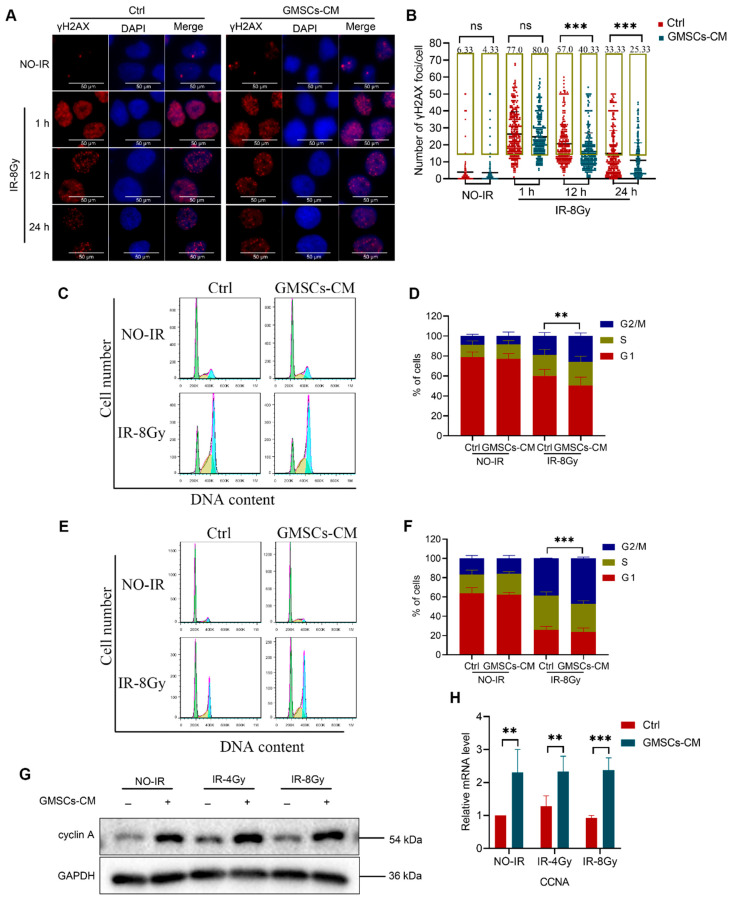
GMSCs enhance the ability of skin cells to repair DNA damage. (**A**,**B**) Skin cells exposed to 8 Gy. The γH2AX foci were counted at 1, 12 and 24 h post-irradiation. (**C**–**F**) Flow cytometry histograms showing cell cycle progression of skin cells at 24 and 48 h post-irradiation (**C**,**E**). Proportions of cells in each phase of the cell cycle of skin cells at 24 and 48 h post-irradiation (**D**,**F**). (**G**) Western blot analysis of cyclin A protein expression in skin cells exposed to 8 Gy. (**H**) Measurement of the expression of the CCNA by qPCR analysis. Representative results from one of three independent experiments are shown. Data are expressed as the mean ± standard deviation. Statistical comparisons were made by a two-sample Student’s *t*-test, using the SPSS 25.0 software (IBM Corporation, Armonk, NY, USA). ns, not significant *p* > 0.05, ** *p* < 0.01 and *** *p* < 0.001.

IR directly or indirectly causes severe DNA damage, leading to chromosomal aberrations and genomic instability, ultimately resulting in the loss of cell function and death [41,42]. Several studies have shown that the formation of γH2AX foci plays an important role in the protein recruitment and signal transduction cascade of the DNA damage response [43]. Therefore, formation of γH2AX foci was used as a DNA damage marker to evaluate the influence of GMSCs-CM on the repair of IR-induced DNA damage in HaCaT cells. We performed immunofluorescence analysis experiments to detect γH2AX foci at 1, 12 and 24 h after HaCaT cells were exposed to 8 Gy radiation. Statistical analysis was performed by counting the γH2AX foci in each cell and counting cells with γH2AX foci ≥ 15 as positive cells. The results revealed no significant difference in the mean number of intracellular γH2AX foci between the control and GMSCs-CM treatment groups at 1 h after IR. However, at 12 and 24 h after IR, the average γH2AX foci in the control group were significantly higher than that in the GMSCS-CM group. Counting the positive cells produced the same results (Figure 4A,B). The above experimental results indicated that IR induces DNA damage in HaCaT cells, and GMSCS-CM treatment enhances their ability to repair IR-induced DNA damage.

The DNA repair pathway can restore DNA integrity when the cell cycle is blocked, and cell cycle regulation may be the most important determinant of the sensitivity to IR [44]. Studies have shown that the G2 phase arrest in cells may provide time for the repair process, which is essential to ensure cell survival after sublethal DNA damage [45,46]. The flow cytometric analysis of cell cycle distribution showed that at 24 and 48 h after 8 Gy of IR, the ratio of cells in the G2/M phase was higher than that in the non-irradiated group. Moreover, the proportion of cells in the G2/M phase in the GMSCS-CM treatment group after exposure to IR was significantly higher than that in the control group (Figure 4C–F). We also used cyclin A as a G2/M phase cell cycle marker in the Western blot analysis. The results showed that the level of cyclin A protein in the GMSCs-CM-treated cells was significantly higher than that in the GMSCs-CM untreated group, whether to irradiate or not (Figure 4G). Similarly, the transcription level of cyclin A (CCNA) in the GMSCS-CM-treated cells was also significantly upregulated (Figure 4H). These results indicated that GMSCS-CM induces G2/M phase cell cycle arrest in HaCaT cells after exposure to IR and enhances the ability of repair of IR-induced DNA damage.

### 3.5. GMSCs Regulate IR-Induced Activation of the EGFR/STAT3 Signaling Pathway

Epidermal growth factor receptor (EGFR) signaling plays a role in all aspects of skin physiology and pathology [47,48]. It has been shown to be essential for the proliferation, migration and survival of keratinocytes to maintain normal skin homeostasis or restore epidermal integrity after injury [49]. EGFR signaling is usually limited to the basal layer of the epidermis, where the proliferating cells are located [49]. Signal transducers and activators of transcription 3 (STAT3) are effectors of EGFR signal transduction, previously confirmed to be related to skin remodeling [50]. In addition, other studies have shown that EGFR mediates the migration of keratinocytes, and its activation can induce the phosphorylation of STAT3, which is also involved in the migration and proliferation of keratinocytes [51,52].

**Figure 5:**
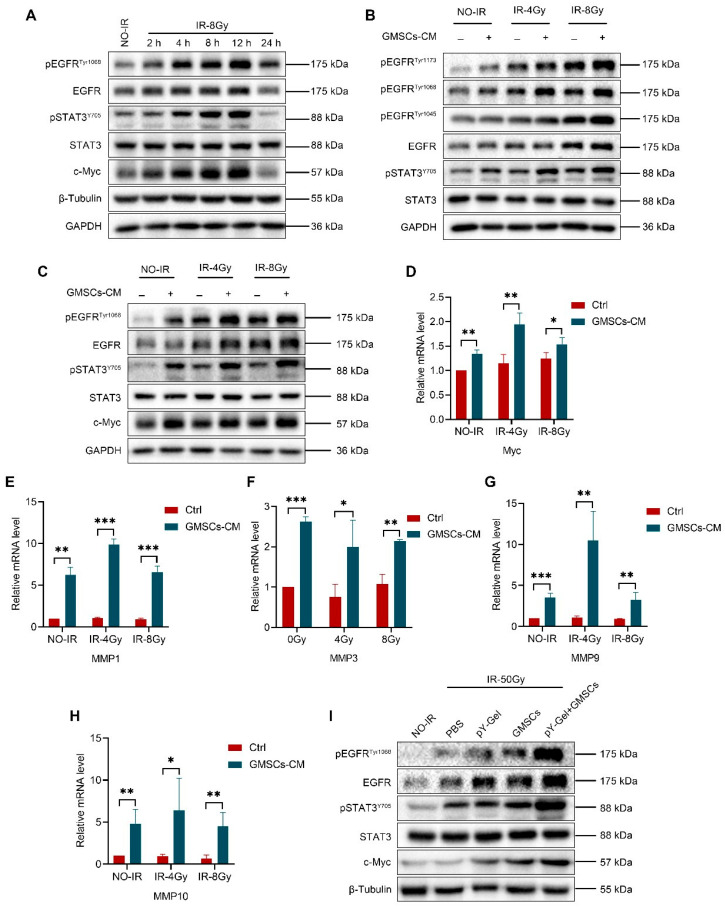
GMSCs regulate IR-induced activation of the EGFR/STAT3 signaling pathway. (**A**) pEGFR^Tyr1068^, EGFR, pSTAT3^Y705^, STAT3 and c-Myc expression levels in IR-treated skin cells for the indicated times. (**B**,**C**) pEGFR^Tyr1173^, pEGFR^Tyr1068^, pEGFR^Tyr1045^, EGFR, pSTAT3^Y705^, STAT3 and c-Myc expression levels in GMSCs-CM-treated skin cells for 6 h after 4 and 8 Gy irradiation. (**D**) Measurement of the expression of Myc by qPCR analysis at 6 h after 4 and 8 Gy irradiation. (**E**–**H**) Measurement of the expression of MMP1, MMP3, MMP9 and MMP10 by qPCR analysis at 6 h after 4 and 8 Gy irradiation. (**I**) pEGFR, EGFR, pSTAT3^Y705^, STAT3 and c-Myc expression levels in pY-Gel+ GMSCs-treated skin tissues for 30 days after 50 Gy irradiation. Representative results from one of three independent experiments are shown. Data are expressed as the mean ± standard deviation. Statistical comparisons were made by two-sample Student’s *t*-test, using the SPSS 25.0 software (IBM Corporation, Armonk, NY, USA). * *p* < 0.05, ** *p* < 0.01 and *** *p* < 0.001.

We hypothesized that GMSCs-CM promotes skin cell repair by regulating the activation of EGFR and STAT3. Western blot analysis was used to evaluate the effect of the activation of the EGFR/STAT3 pathway after exposure of skin cells to IR. The results revealed that the protein levels of pEGFR, EGFR, pSTAT3 and c-Myc increased gradually after exposure to IR, and the trend was consistent and time-dependent. The protein levels decreased significantly 24 h after IR, and the trend was the same, but exposure to IR had no significant effect on the total protein level of STAT3 (Figure 5A and Supplementary Figure S3A). Together, these results showed that exposure to IR can induce the activation of the EGFR/STAT3 pathway in skin cells. Thus, we verified whether GMSCs-CM regulates the IR-induced activation of the EGFR/STAT3 signaling pathway in skin cells. The results revealed that the protein levels of pEGFR, EGFR, pSTAT3 and c-Myc were increased in HaCaT cells after exposure to IR and significantly elevated in the GMSCS-CM-treated HaCaT cells compared with the untreated control HaCaT cells (Figure 5B,C and Supplementary Figure S3B,C). Furthermore, the expression level of Myc in the GMSCs-CM-treated HaCaT cells was also significantly increased compared with the untreated control cells (Figure 5D). We also tested the effects of GMSCs-CM treatment on the expression of downstream target genes of EGFR/STAT3 in HaCaT cells after exposure to IR. Matrix metalloproteinases (MMPs) are related to angiogenesis and cell migration mechanisms and are known to be an important part of epithelial repair [53]. Our results revealed significantly increased expression levels of the target genes MMP1, MMP3, MMP9 and MMP10 (Figure 5E–H) in the GMSCs-CM-treated cells compared with the untreated control cells. These results suggest that GMSCS-CM regulates the IR-induced activation of the EGFR/STAT3 signaling pathway in skin cells. We further verified whether GMSCs regulate the IR-induced activation of the EGFR/STAT3 signaling pathway in skin tissue. The results showed that the protein levels of pEGFR, EGFR, pSTAT3 and c-Myc were increased in irradiated skin tissues compared with the non-irradiated tissues. Compared with the other groups, the phosphorylated protein levels in the pY-Gel+ GMSCs group were significantly increased (Figure 5I and Supplementary Figure S3D). These results indicated that treatment with pY-Gel-encapsulated GMSCs regulates the IR-induced activation of the EGFR/STAT3 signaling pathway in skin tissues.

### 3.6. GMSCs Affect Cell Radiosensitivity by Regulating the EGFR/STAT3 Signaling Pathway

**Figure 6:**
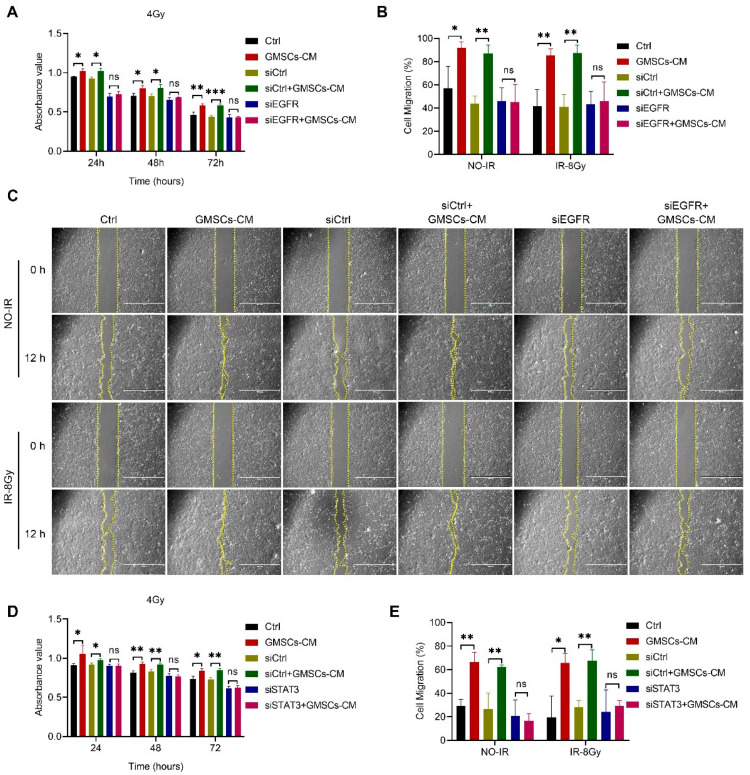
GMSCS-CM promoted the proliferation and migration of skin cells through the induction of phosphorylation and activation of EGFR/STAT3. (**A**) CCK-8 assay of HaCaT cell proliferation following knockdown of EGFR. (**B**,**C**) Evaluation of the migration ability of HaCaT cell following knockdown of EGFR by the scratch wound assay. (**D**) CCK-8 analysis of HaCaT cell proliferation following knockdown of STAT3. (**E**) Quantitative analysis of the migration rates. Scale bars, 1000 μm. Representative results from one of three independent experiments are shown. Data are expressed as the mean ± standard deviation. Statistical comparisons were made by two-sample Student’s *t*-test, using the SPSS 25.0 software (IBM Corporation, Armonk, NY, USA). ns, not significant *p* > 0.05, * *p* < 0.05, ** *p* < 0.01 and *** *p* < 0.001.

It has been shown that inhibition of EGFR and STAT3 phosphorylation suppresses keratinocyte proliferation and migration [52]. To further examine whether GMSCs promote the proliferation and migration of skin cells through the activation of the EGFR/STAT3 signaling pathway, we used three kinds of siRNAs, designated as #1, #2 and #3, to knock down EGFR expression in HaCaT cells and measured the EGFR protein level by Western blot analysis (Supplementary Figure S4A). We randomly selected siEGFR#2 for the subsequent experiments. The results revealed that the cell proliferation capacity of the siEGFR group decreased, and GMSCs-CM treatment had no significant effect on cell proliferation in the siEGFR group (Figure 6A and Supplementary Figure S4B). The scratch test results showed that the cell migration capacity of the cells in the siEGFR group was decreased, and the GMSCS-CM treatment had no significant effect on the cell migration capacity of the siEGFR group (Figure 6B,C). These results demonstrated that EGFR knockdown reduced the proliferation and migration capacity of HaCaT cells, and the ability of GMSCS-CM to promote the proliferation and migration of HaCaT cells disappeared. In other words, GMSCS-CM promotes the proliferation and migration of skin cells through the activation of EGFR.

Additionally, it has also been shown that interference with STAT3 expression in epidermal keratinocytes impairs the wound healing process [54]. We knocked down STAT3 in the same way and verified (Supplementary Figure S4C). The results showed that the proliferation capacity of cells in the siSTAT3 group was reduced, and the GMSCS-CM treatment had no significant effect on the proliferation capacity of the cells in the siSTAT3 group (Figure 6D and Supplementary Figure S4D). The scratch test results showed that the cell migration ability of the siSTAT3 group was reduced, and the GMSCS-CM treatment had no significant effect on the migration ability of the cells in the siSTAT3 group (Figure 6E and Supplementary Figure S4E). These results suggested that STAT3 knockdown reduced the proliferation and migration ability of HaCaT cells and impaired the ability of GMSCS-CM to promote cell proliferation and migration. In other words, GMSCS-CM promoted the proliferation and migration of skin cells by activating STAT3.

## 4. Discussion

Since radiation-induced skin wounds/dermatitis is one of the leading factors that threaten the physical and mental health of patients and cause an economic burden [1], the research and development of an ideal treatment method is both important and urgent. In the present study, we effectively used a combination of Nap-GDFDFpDY (pY-Gel) self-assembling peptide hydrogel-encapsulated GMSCs to promote skin defect healing in a mouse model of radiation skin injury.

Previous studies showed that the application of mesenchymal stem cells (MSCs)-laden hydrogels promotes improved wound healing [55]. Among odontogenic MSCs, GMSCs are readily obtained from the oral cavity and can usually be obtained from discarded biological samples [31]. Several studies have shown that GMSCs can accelerate skin wound healing by enhancing re-epithelialization, collagen deposition, angiogenesis, inhibiting the production of inflammatory cytokines and increasing anti-inflammatory cytokines [56]. These findings indicate that GMSCs play a key role in skin wound healing. However, to the best of our knowledge, the ability of GMSCs to repair radiation-induced skin damage has not been reported. Cell-laden hydrogels are widely used in tissue engineering and regenerative medicine [55,57]. As such, this study used a self-assembled polypeptide supramolecular hydrogel as the carrier of GMSCs to treat radiation-induced skin injury.

Wound healing is a dynamic process which involves a coordinated effort by multiple biological pathways. MSCs therapy can influence each of these stages of tissue repair, improving wound healing [22]. More recently, methods to enhance the effectiveness of MSCs by the use of hydrogel scaffolds have also been tested in treating skin damage and have shown great promise [55,58]. Our in vivo study showed that the severity of the radiation skin damage in the pY-Gel+ GMSCs group was reduced, and the skin wound repair ability was enhanced compared with that in other irradiated mice. In addition, the skin histological analysis of mice showed that the radiation-induced skin fibrosis was significantly reduced in the pY-Gel+ GMSCs group, and the tissue structure of the skin tended to be normal. These results indicate that the treatment with pY-Gel self-assembling peptide hydrogels for GMSCs has positive effects on re-epithelialization and remodeling. At the same time, we also found that the treatment with pY-Gel self-assembling peptide hydrogels for GMSCs partially restores the proliferation capacity of epidermal cells, which have a positive impact on wound healing. All these factors can promote the healing of radiation-induced skin damage. Since the healing effect on the pY-Gel+ GMSCs group was faster than that on the pY-Gel and GMSCs treatment groups, we hypothesized that these results had two major implications. First, GMSCs could promote the healing of radiation-induced skin wounds. Second, pY-Gel provided a suitable environment for GMSCs to exert their biological effects. Considering the availability of GMSCs and their ability to repair skin damage, GMSCs are unique and promising candidates for skin tissue engineering in the appropriate microenvironment. These results indicate that the pY-Gel self-assembling peptide hydrogels for GMSCs intervention may be a promising option for the treatment of radiation-induced skin injury.

Although stem cell-based therapies have shown beneficial effects on wound healing and tissue regeneration, it has been reported that the predominant mechanism by which stem cells repair tissue is paracrine action [22,59]. Some studies showed that conditioned mediums contained high concentrations of growth factors and cytokines associated with angiogenesis and endothelial cell migration [60,61]. Increasing evidence supports that MSCs-CM significantly accelerated wound closure and enhanced the wound healing quality [62]. As such, we chose to use GMSCs-CM for in vitro studies. It has been reported that MSCs-CM effectively improves wound healing quality by secreted factors that promoted HUVEC proliferation, regeneration of sebaceous glands and angiogenesis [62]. Another study also demonstrated that MSCs-CM could promote wound repair and skin regeneration via improvement of cellular behaviors of fibroblasts in the microenvironment [63]. Our results showed that GMSCs-CM promotes the proliferation and migration of skin cells. In addition, analysis of cell cycle arrest, which provides sufficient time for DNA damage repair, thus maintaining the stability of the cell genome [64], revealed several interesting findings. We found, through cell cycle and immunofluorescence assays, that skin cells treated with GMSCS-CM were arrested in the G2/M phase after irradiation. As a result, the ability to repair DNA damage was enhanced. This suggests that GMSCs-CM may secrete various factors to stimulate fibroblasts and keratinocyte proliferation, migration and DNA damage repair to enhance injured skin regeneration and functional recovery.

Since EGFR and STAT3 are involved in the migration and proliferation of keratinocytes [42], stimulating EGFR can lead to the phosphorylation, and thus activation, of STAT3 [52]. In this study, we found that exposure to IR could activate the EGFR/STAT3 signaling pathway and further evaluated the influence of GMSCS-CM on the major molecules in this pathway. The experimental results showed that GMSCs regulate the phosphorylation level of EGFR and STAT3, as well as the downstream effector c-Myc in skin cells, after exposure to IR. Moreover, our in vivo experiments showed similar results. The Designer Antimicrobial Peptide A-hBD-2 has been reported to increase keratinocyte migration and proliferation through phosphorylation of EGFR and STAT3 and to inhibit keratinocyte terminal differentiation to promote wound healing in vivo [65]. In this study, the siRNA-mediated knockdown of EGFR and STAT3 led to the inhibition of the migration and proliferation of skin cells, indicating that GMSCs-CM induces the phosphorylation of EGFR and STAT3, which leads to their activation, thereby mediating skin cell migration and proliferation. Furthermore, IR induced the upregulation of EGFR expression and promoted the phosphorylation of EGFR, which in turn might promote the activation of the STAT3 and the transcription of its downstream genes. In particular, phosphorylated STAT3 can result in more efficient DNA damage repair and better survival of cells [66]. In our studies, IR induces DNA damage in HaCaT cells and GMSCS-CM treatment enhances their ability to repair IR-induced DNA damage. Collectively, we speculate that GMSCs-CM significantly promotes the DNA repair and survival of skin cells via upregulating pEGFR and pSTAT3 expression levels after irradiation.

This study provided a novel concept for the application of GMSCs. Since it has been proven that GMSCs play a critical role in skin tissue repair and regeneration [30,66], some researchers began to explore new methods to apply GMSCs in the tissue repair field. Shi et al. showed that the incorporating of GMSC-derived exosomes to hydrogel could effectively promote healing of diabetic skin defects [25]. Our study suggests that GMSCs-CM can promote skin cell proliferation and migration by activating the EGFR/STAT3 signaling pathway in vitro; while pY-Gel supramolecular hydrogel associated to GMSCs can promote the damage repair of radioactive skin tissue in vivo. Furthermore, increasing evidence supports that GMSCs secrete various growth factors and chemokines for promoting wound healing through paracrine effects [25,30,67]. Conditioned media loaded in hydrogel may have significant potential as a novel alternative to whole cell-based therapy and to achieve “cell-free regenerative medicine”.

In summary, we highlight the profound effect of pY-Gel supramolecular hydrogel on skin damage repair and demonstrate that GMSCs-CM may promote skin cell proliferation and migration through activation of the EGFR/STAT3 signaling pathway, which ultimately promotes skin injury repair. Therefore, GMSCs-mediated skin damage repair sheds a light on the cell-based treatment for radiation-induced skin damage. In short, the pY-Gel self-assembling peptide hydrogels for GMSCs may become a good option for clinical application in the treatment of radiation-induced skin injury.

## Data Availability

No new data were created or analyzed in this study. Data sharing is not applicable to this article.

## Supplementary Materials

The following supporting information can be downloaded at: https://www.mdpi.com/article/10.3390/cells11193089/s1, Figure S1: Gel-forming process of pY-Gel self-assembled hydrogel-encapsulated GMSCs; Figure S2: Inflammatory reactions in skin tissues and cells; Figure S3: GMSCs regulate IR-induced activation of the EGFR/STAT3 signaling pathway; Figure S4: GMSCS-CM promoted the proliferation and migration of skin cells through the induction of phosphorylation and activation of EGFR/STAT3; Table S1: Sequences of the primers used for qRT-PCR; Table S2: Sequences of the siRNAs for RNA interference.

## Abbreviations

CRI: cutaneous radiation injury; GMSCs: gingiva mesenchymal stem cells; pY-Gel: Nap-GDFDFpDY; CM: conditioned medium; IR: ionizing radiation; MSCs: mesenchymal stem cells; BMMSCs: bone marrow mesenchymal stem cells; Y-Gel: Nap-GDFDFDY; FBS: fetal bovine serum; SPF: specific-pathogen free; AEWC: Animal Ethical and Welfare Committee; H&E: hematoxylin and eosin; SDS-PAGE: sodium dodecyl sulfate–polyacrylamide gel electrophoresis; PVDF: polyvinylidene fluoride; CCK-8: Cell Counting Kit-8; PI: propidium iodide; siRNAs: small interfering RNAs; EGFR: epidermal growth factor receptor; STATs: signal transducers and activators of transcription; MMPs: matrix metalloproteinases; ECM: extracellular matrix.
