# Supplementary material for: Supramolecular Hydrogel-Wrapped Gingival Mesenchymal Stem Cells in Cutaneous Radiation Injury
Source: Cells. 2022 Sep 30;11(19):3089. doi: 10.3390/cells11193089 (PMC9564043; doi:10.3390/cells11193089)
Supplement: Supplementary file 1 [file cells-11-03089-s001.zip › cells-1899301-supplementary.pdf]

cells

Supplementary Data

Table S1. Sequences of the primers used for qRT-PCR.

Gene	Forward sequences	Reverse sequences
Human CCNA	5-GGAGACCTTTTACTTGGCACA-3	5-TCTGTCACATACGCAAACTGG-3
Human Myc	5- TGAGGAGACACCGCCCACCA-3	5- AGACTCTGACCTTTTGCCAGGA-3
Human MMP1	5- GCGACTCTAGAAACACAAGAGC-3	5- GCCCAAAGAATTCCTGCAT-3
Human MMP3	5- GACACCAGCATGAACCTTG-3	5- GAACTTCTGCATTTCTCGGAT-3
Human MMP9	5- TGTGCTCTTCCCTGGAGACCT-3	5- CAGGCCCCAGAGATTTCGACT-3
Human MMP10	5- TTTCTGCATTTTGGCCCTC-3	5- CCTCTTGGATAACCTGCTT-3
Human GAPDH	5- TCTGACTTCAACAGCGACACC-3	5-CTGTTGCTGTAGCCAAATTCGTT-3
Human IL6	5- ACAAATTCGGTACATCCTCGAC-3	5- TGATGATTTTCACCAGGCAAG-3
Human IL1β	5- CCTGCCCACAGACCTTCCAG-3	5- ACATAAGCCTCGTTATCCCAT-3
Human TNFα	5- GATTCTGAGCAAAATAGCCAGCA-3	5- GGCTTCCTTCTTGTTGTGTGT-3
Mouse IL6	5-CCGTGTGGTTACATCTACCCT-3	5- CGTGGTTCTGTTGATGACAGTG-3
Mouse IL1β	5- GCAACTGTTCCTGAACTCAACT -3	5- ATCTTTTGGGGTCCGTCAACT-3
Mouse TNFα	5-CTGGATGTCAATCAACAATGGGA-3	5-ACTAGGGTGTGAGTGTTTTCTGT-3
Mouse GAPDH	5-AGGTCGGTGTGAACGGATTTG-3	5-TGTAGACCATGTAGTTGAGGTCA-3

Table S2. Sequences of the siRNAs for RNA interference.

Gene	Forward sequences	Reverse sequences
STAT3#1	5- GCAACAGAUUGCCUGCAUUTT-3	5- AAUGCAGGCAAUCUGUUGCTT-3
STAT3#2	5- CCCGGAAAUUUAACAUUCUTT-3	5- AGAAUGUUAAAUUUCCGGGTT-3
STAT3#3	5- GCUGAACAACAUGUCAUUUTT-3	5- AAAUGACAUGUUGUUCAGCTT-3
EGFR#1	5- GCAGAGGAAUUAUGAUCUUTT-3	5- AAGAUCAUAAUUCCUCUGCTT-3
EGFR#2	5- GCAACAUGUCGAUGGACUUTT-3	5- AAGUCCAUCGACAUGUUGCTT-3
EGFR#3	5- GGAGAUAAGUGAUGGAGAUTT-3	5- AUCUCCAUCACUUAUCUCCTT-3

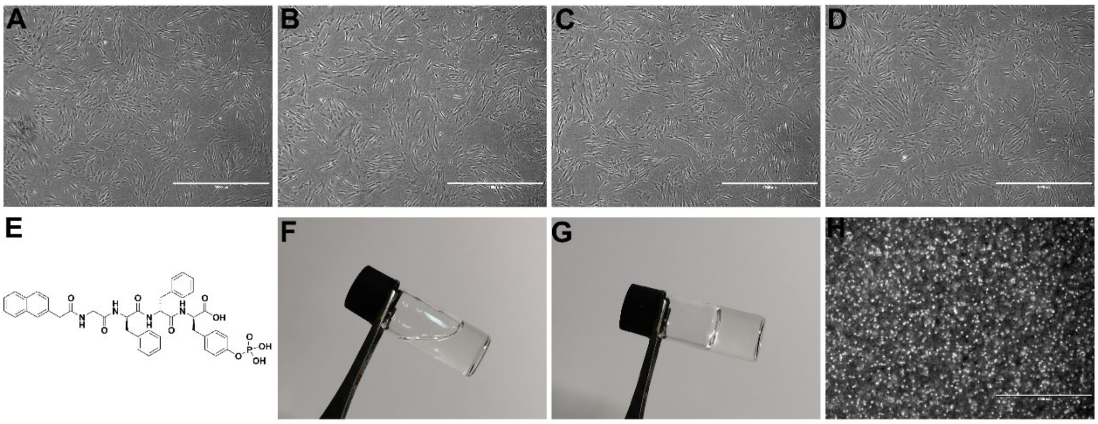

Figure S1. Gel-forming process of pY-Gel self-assembled hydrogel-encapsulated GMSCs. A-D. The morphological changes of GMSCs from the passage 3 to passage 6 (P_3-P_6) were observed under a microscope. E. Chemical structure of pY-Gel. F-G. Optical image of the structure formed by the pY-Gel. The clear viscous solution (F) forms gel (G) at room temperature. H. Optical image of the suspension growth of GMSCs in the pY-Gel. Scale bars, 1,000 μm.

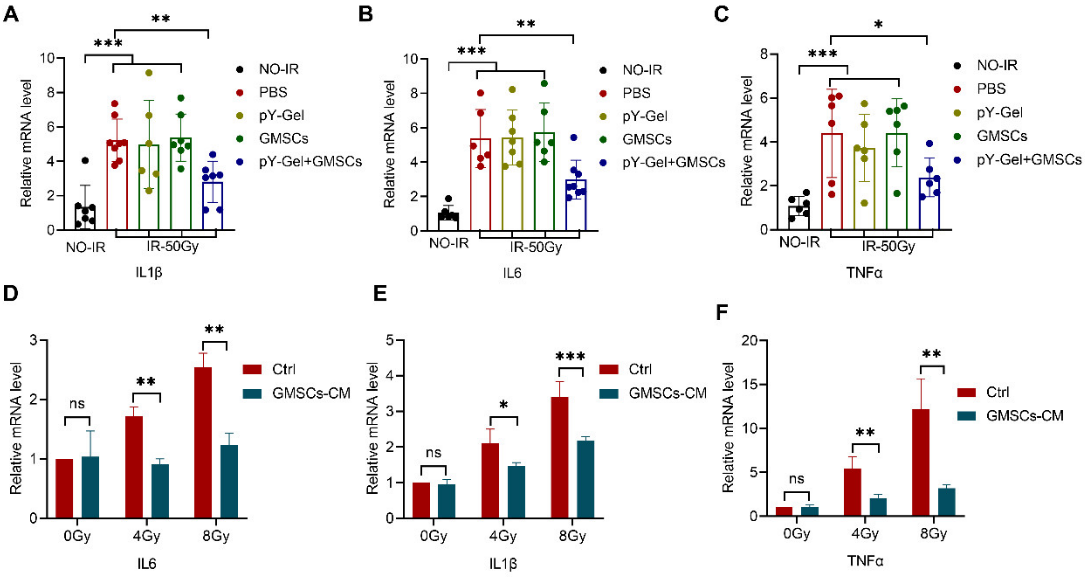

Figure S2. Inflammatory reactions in skin tissues and cells. A-C. Measurement of the expression of the indicated cytokines by qPCR analysis. n=5. IL1β=interleukin-1β; IL6=interleukin-6; TNFα=tumor necrosis factor alpha. Data are expressed as the mean ± standard deviation. Statistical comparisons were made by ANOVA for multiple comparisons. D-F. Measurement of the expression of the IL6, IL1β, and TNFα by qPCR analysis at 6 h after 8 Gy irradiation. Representative results from one of three independent experiments are shown. *$p < 0.05$, **$p < 0.01$ and ***$p < 0.001$.

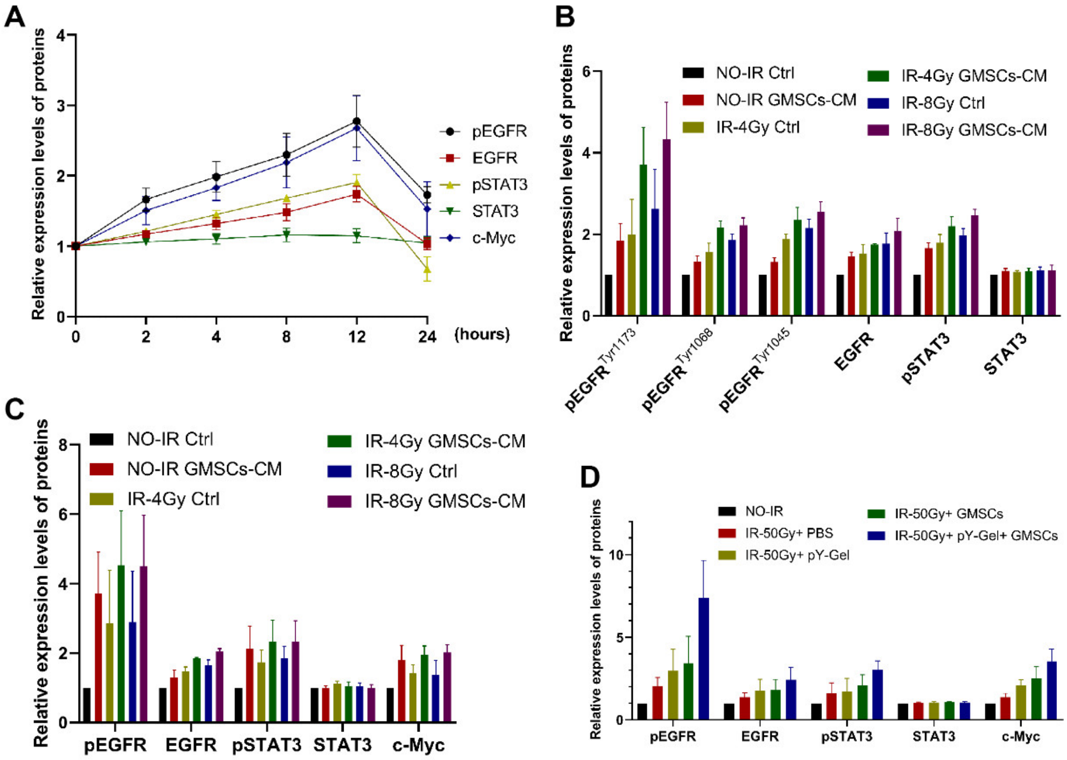

Figure S3. GMSCs regulate IR-induced activation of the EGFR/STAT3 signaling pathway. A. Quantification of the pEGFR^{Tyr1068}, EGFR, pSTAT3^{Y705}, STAT3, and c-Myc band intensity by ImageJ in IR-treated skin cells for the indicated times. The protein level of cells in the NO-IR group was normalized to 1. B-C. Quantification of the pEGFR^{Tyr1173}, pEGFR^{Tyr1068}, pEGFR^{Tyr1045}, EGFR, pSTAT3^{Y705}, STAT3 and c-Myc band intensity by ImageJ in GMSCs-CM-treated skin cells for 6 h after 4 and 8 Gy irradiation. The protein level of cells in the NO-IR group was normalized to 1. D. Quantification of the pEGFR, EGFR, pSTAT3^{Y705}, STAT3 and c-Myc band intensity by ImageJ in pY-Gel+ GMSCs-treated skin tissues for 30 days after 50 Gy irradiation. The protein level of skin tissue in the NO-IR group was normalized to 1.

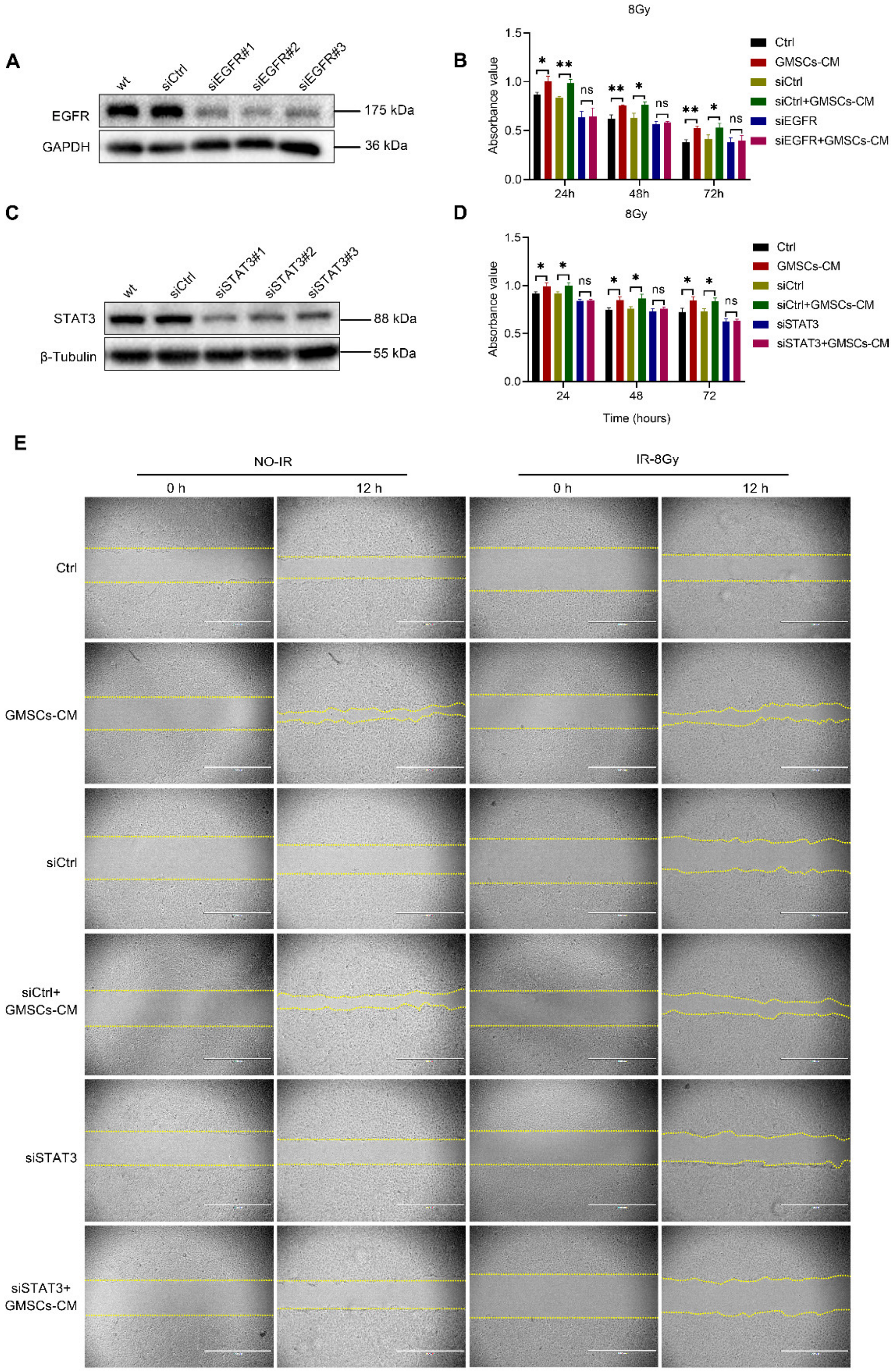

Figure S4. GMSCS-CM promoted the proliferation and migration of skin cells through the induction of phosphorylation and activation of EGFR/ STAT3. A. Western blot analysis of EGFR levels in skin cells following knockdown of EGFR. B. CCK-8 assay of HaCaT cell proliferation following knockdown of EGFR. C. Western blot analysis of STAT3 levels in skin cells following knockdown of STAT3. D. CCK-8 analysis of HaCaT cell proliferation following knockdown of STAT3. E. Evaluation of the migration ability of HaCaT cells following knockdown of STAT3 by the scratch wound assay. Scale bars, 1,000 μm. Representative results from one of three independent experiments are shown. $*p < 0.05$, $**p < 0.01$ and $***p < 0.001$.
